# The effectiveness and safety of intracavernosal botulinum toxin injections in the management of erectile dysfunction: a systematic review and meta-analysis of clinical studies

**DOI:** 10.1093/sexmed/qfaf034

**Published:** 2025-05-06

**Authors:** Karl H Pang

**Affiliations:** Division of Surgery and Interventional Science, University College London, London, United Kingdom; Department of Urology, Chelsea and Westminster Hospital NHS Foundation Trust, London, United Kingdom

**Keywords:** erectile, dysfunction, intracavernosal injection, botulinum neurotoxin

## Abstract

**Background:**

Patients with erectile dysfunction (ED) are often left with limited nonsurgical options when conventional pharmacotherapies become ineffective. Botulinum neurotoxin serotype A (BoNT-A) intracavernosal injections (ICIs) have been demonstrated in a few clinical studies to be effective and safe in managing ED and may serve as a first- or second-line option.

**Aim:**

To perform a systematic review and meta-analysis on the effectiveness and safety of BoNT-A ICI in managing ED with a focus on human studies.

**Methods:**

A systematic review and meta-analysis of clinical studies was performed in accordance with the PRISMA 2020 statement. Adult men with ED who received BoNT-A were included in the study. The risk of bias assessment of included studies was performed using the JBI assessment checklists.

**Outcomes:**

The effectiveness and safety of BoNT-A ICI. Measures of effectiveness included the increase or change in International Index for Erectile Function (IIEF-EF) score/Sexual Health Inventory for Men (SHIM) score/Erection Hardness Score (EHS) or change in Doppler ultrasound parameters and the percentage of patients achieving the minimal clinically important difference (MCID) score.

**Results:**

The search retrieved 61 articles, and 2 randomized controlled trials (RCTs) and 4 retrospective studies met the inclusion criteria. Three types of BoNT-A were evaluated: onaBoNT-A, aboBoNT-A, and incoBoNT-A. At least 40% of the patients achieved the MCID (2-7 points increase in the IIEF-EF/SHIM score) depending on the severity of ED) at ≥1 month. When directly comparing all 3 BoNT-A, the overall response rate was 77.5%, with no statistically difference between the 3 types. For aboBoNT-A, 500 U appeared to sustain the effectiveness longer compared to 250 U. OnaBoNT-A improved peak systolic velocity on Doppler ultrasound compared with saline. Meta-analysis of the 2 RCTs demonstrated a mean difference of 4.35 (*P* = .008) in the SHIM score at 12 weeks in favor of onaBoNT-A over normal saline. No systemic side effects have been reported, and the only infrequent local side effect was transient penile pain. Only one case of priapism was reported.

**Strengths and Limitations:**

This is the first systematic review summarizing data from human studies on ED and BoNT-A ICI. The inclusion criteria and outcomes of the included studies varied, which limited the number of studies included and parameters assessed in the meta-analysis.

**Conclusion:**

BoNT-A may become an acceptable non-surgical option. However, there is a lack of clinical randomized or observational studies on this topic, and more randomized studies with standardized reporting are required to replicate current results and investigate the long-term benefits of BoNT-A as a monotherapy or adjunct therapy in the management of ED.

## Introduction

The management options for erectile dysfunction (ED) include lifestyle modifications, vacuum devices, oral phosphodiesterase-5 inhibitors (PDE5i), intraurethral alprostadil, intracavernosal injections (ICIs), low-intensity shockwave therapy (Li-SWT), and penile prosthesis (PP).^1^^,^^2^ For patients who do not want to undergo PP surgery, are not fit for surgery, or who are unresponsive or intolerant to pharmacotherapies, treatment options become limited. While Li-SWT is a non-surgical option for vasculogenic ED only, long-term outcome data are lacking and treatment regimen is not standardized, requiring ongoing research.^3^^,^^4^

Alternative regenerative and novel treatments are in development and have been tested in clinical trials. These treatments include ICI of stem cells,^5^ platelet-rich plasma (PRP),^6^ and botulinum neurotoxin serotype A (BoNT-A).^7^ Although clinical studies on their effectiveness and safety are promising, there is still insufficient robust evidence to recommend their use outside of clinical trial settings.

There are 3 types of BoNT-A: onabotulinum toxin A, incobotulinum toxin A, and abobotulinum toxin A. All have been used in different medical and surgical specialties. In urology, the treatment is approved for idiopathic^8^ or neurogenic detrusor overactivity^9^ via intradetrusor injections. BoNT-A inhibits the release of acetylcholine and norepinephrine with the goal of relaxing muscles.^7^ Therefore, BoNT-A may have a role in modulating cavernosal smooth muscle function.

Although BoNT-A is not considered a salvage therapy, it provides an option as a first-line treatment for men who experience side effects from PDE5i or as a second-line alternative therapy to ICI prostaglandins in those who are unresponsive to PDE5i. Several retrospective and randomized-controlled studies published in the past 5 years have shown promising results. However, there are differences in the types of toxins and doses used and the outcome measures reported. The aim of this systematic review is to summarize the current clinical evidence on the effectiveness and safety of BoNT-A in the management of ED.

## Methods

The PRISMA 2020 statement (Table S1)^10^ was used as a guide for this systematic review. A search was performed on PubMed on February 2, 2025, using the following terms: (erectile dysfunction) AND (Botox OR BoNT-A OR Xeomin OR Dysport OR botulinum OR neurotoxin). A population (P), intervention (I), comparator (C), outcome (O), study (S) framework was used to determine the eligibility of studies: P, adult men with ED; I, BoNT-A; C, any oral, injectable, or non-surgical treatment comparisons, or placebo; O, any applicable outcome measures such as validated or nonvalidated questionnaires, Doppler ultrasound scan (USS), and complications; S, any study designs including randomized controlled trials (RCTs), prospective or retrospective studies, case series, and case reports. Animal studies and reviews were excluded.

### Data extraction and analysis

Data extracted included the study design, inclusion characteristics, number of patients, BoNT-A details (type of toxin, dose, injection location, and frequency), preintervention details (questionnaire scores and Doppler USS findings), follow-up intervals, assessment outcomes, and complications.

Meta-analyses were performed using Review Manager 5.4.1 (The Cochrane Collaboration), and the results were presented in the form of forest plots. Inverse variance weighted mean difference with 95% confidence intervals (95% CI) was used as a summary measure for continuous variables. If studies reported the median and interquartile ranges (IQRs), estimation of the mean and SD was performed using Abbas et al.’s meta-analysis accelerator.^11^ Statistically significance was defined as *P*-value <.05. Pooled estimates were calculated using the random-effect model for all outcome variables due to heterogeneity between the included studies. Study heterogeneity was quantified using the chi-squared and *I*^2^ statistics.

The risk of bias (RoB) of included studies was assessed using the JBI critical appraisal checklists.^12^

## Results

**Figure 1:**
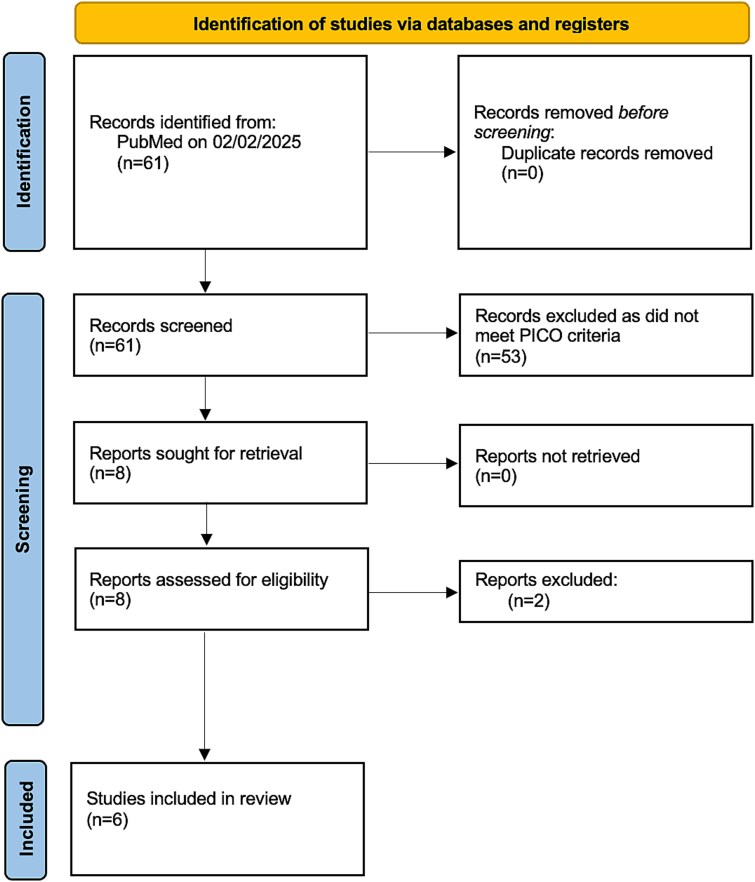
PRISMA 2020 flowchart for the current systematic review.

The search retrieved 61 articles (Figure 1). After screening abstracts and full-text article, 6^13-18^ studies fulfilled the predefined PICOS criteria and were included for analysis. There were 2 RCTs^16^^,^^17^ and 4 retrospective studies.^13-15^^,^^18^ All 4 retrospective studies were from the same institution, conducted by Giuliano et al. The baseline characteristics, inclusion criteria, and outcomes of each study are summarized in Tables 1 and 2. The RoB assessment of each study using the JBI checklists is summarized in Table S2.

**Table 1:** Characteristics of included studies.

**Author**	**Country**	**Design**	**Inclusion and exclusion criteria**	**Investigation**	**Intervention, n**	**Control, *n***	**Intervention ICI**	**Control ICI**	**Protocol**
Giuliano, 2023	France	Retrospective	– Age > 18 y with ED (IIEF-EF <26) – Insufficient response to PDE5i or PGE1 ICI for at least 3 m – Received >2 BoNT ICI	Effectiveness and safety of repeated BoNT in men with ED and insufficient response to PDE5i or PGE1 ICI	85	NA	OnaBoNT-A 100 U, IncoBoNT-A 100 U, or AboBoNT-A 500 U	NA	– Penile loop ring at base for 30 min – 0.5 mL BoNT into each CC – At least 5 m between injections at individual’s request
Giuliano, 2022a	France	Retrospective	– Age > 18 y with ED – Insufficient response to PDE5i or PGE1 ICI for at least 3 m – Received >1 incoBoNT-A ICI	Safety and effectiveness of incoBoNT-A ICI as an add-on therapy to PDE5i or PGE1 ICI to treat ED that did not respond sufficiently to this treatment alone.	54	NA	IncoBoNT-A100U	NA	– Penile loop ring at base for 30 min – 0.5 mL (50 U) BoNT into each CC – At least 3 m between injections at individual’s request
Giuliano, 2022b	France	Retrospective	– Age > 18 y with ED (IIEF-EF <26) – Insufficient response to PDE5i or PGE1 ICI	Effectiveness and safety of combined treatment involving a single injection of BoNT ICI as an add-on therapy to PDE5i or PGE1 ICI.	123	NA	OnaBoNT-A 100 U or aboBoNT-A 250 U or 500 U	NA	– Penile loop ring at base for 30 min – 0.5 mL BoNT into each CC
Abdelrahman, 2022	Egypt	RCT	– Age > 21 y with ED – Refractory to PDE5i	Safety and efficacy of ICI BoNT in patients with ED refractory to PDE5i.	35	35	OnaBoNT-A 100 U	2 mL normal saline	– Tourniquet at base for 20 min – 2 mL (100 U or saline) distributed at 4 points, right and left distal and proximal shaft
El-Shaer, 2021	Egypt	RCT	– Age 40-70 y with vasculogenic ED – No response to PDE5i or ICI Trimix Exclusion: significant cardiovascular disease, unstable psychiatric conditions, anatomical/hormonal/neurological abnormalities, and radical pelvic surgery.	Safety, efficacy, and durability of different ICI onaBoNT-A doses in the management of vasculogenic ED nonresponding to pharmacological therapy.	121	55	OnaBoNT-A 50 U or 100 U	1 mL normal saline	– Rubber band at root for 20 min – 1 mL (50 U or 100 U or saline) distributed at 4 points, right and left distal and proximal shaft
Giuliano, 2019	France	Retrospective	– Age > 18 y with ED (IIEF-EF <26) – Insufficient response to PDE5i or PGE1 ICI Exclusion: contraindication to BoNT-A	Safety and efficacy of aboBoNT-A as add-on therapy to PDE5i or PGE1 ICI for the treatment of adult men with ED.	47	NA	AboBoNT-A 250 or 500 U	NA	– Penile loop ring at base for 30 min – 0.5 mL (250 or 500 U) BoNT into each CC

Abbreviations: BoNT, botulinum neurotoxin; CC, corpus cavernosum; ED, erectile dysfunction; IIEF, International Index for Erectile Function; ICI, intracavernosal injection; m, month; NA, not application; PDE5i, phosphodiesterase-5 inhibitor; PGE1, prostaglandin E1; y, year.

**Table 2:** Outcome measures and side effects of included studies.

				**Outcome measure**								
**Author**	**Pre-BoNT EF**	**Precontrol EF**		**Time between injections**	**Post-BoNT EF**		**Postcontrol EF**	**Change in EF with BoNT**	**BoNT vs control**	**Response rate, *n* (%)**	**Side effects, *n* (%)**	**FU**
Giuliano, 2023	Median (IQR) IIEF-EF: 14.5 (9-19).	NA		Median (IQR): 8.7 m (6.9-12.2).	– Changes in IIEF-EF score similar across repeated injections – Postinjection EF domain score tended to increase.		NA	NR	NA	– MCID: mild ED, 2 points; moderate. 5 points; severe, 7 points – 77.5% achieved MCID - 85.7% in men with mild, 79% for moderate, and 64.3% for severe ED – No significant difference between types of BoNT – Increased with repeated injections: second 67.5%, third 87.5%, and fourth 94.7% injection – Median effectiveness duration: 9 m.	– Penile pain: 4 (4.7) – Burn: 1 (1.2).	2 and 7 m postinjection.
Giuliano, 2022a	Median (IQR) IIEF-EF: 13 (8-19)	NA		Median (IQR): 8.2 m (7-10)	– Median (IQR) IIEF-EF: 21 (12-26) – Responders: 26 (22-29)		NA	Median (IQR) increase in responders: 8 (6-15) points	NA	– MCID: mild ED, 2 points; moderate. 5 points; and severe, 7 points – 55.2% achieved MCID at first FU, 41% at second FU – 76% who received a second injection responded	– Penile pain: 3 (5.6)	2 and 7 m postinjection
Giuliano, 2022b	– Median (IQR) IIEF-EF in all men: 11 (6-17) – IIEF-EF in responders: 17 (10-19)	NA		NA	– Median (IQR) IIEF-EF in responders: 26 (22-28)		NA	NR	NA	– MCID: mild ED, 2 points; moderate, 5 points; and severe, 7 points – 55.2% achieved MCID at 34 (27-42) days, 41% at 5.9 (3.9-8.1) m – 90% in men with mild, 50% for moderate, 33% for severe ED – No significant difference between types of BoNT	– Penile pain: 2 (1.6)	2 and 7 m postinjection
Abdelrahman, 2022	– Mean (SD) SHIM with BoNT: 5.4 (1.7) – EHS: 2.3 (0.6) – PSV: 34.4 (12.2) – EDV: 3.5 (3.7)	– Mean (SD) SHIM with saline: 5.7 (1.1) – EHS: 2.1 (0.5) – PSV: 31.3 (15.6) – EDV: 4.5 (3.4)		NA	– Mean (SD) SHIM with BoNT at 2w: 6.7 (2.2), *P* = .001 – 6w: 10.0 (5.9), *P* < .001 – 12w: 8.3 (4.1), *P* < .001 – EHS at 2w: 2.9 (0.8), *P* < .001 – PSV at 2w: 45.8 (12.2), *P* < .001 – EDV at 2w: 1.7 (3.5), *P* = .2		– Mean (SD) SHIM with saline at 2w: 6.1 (2.8), *P* = .4 – 6w: 5.8 (1.8), *P* = .7 – 12w: 5.6 (1.4), *P* = .2 – EHS at 2w: 2.2 (0.6), *P* = .08 – PSV at 2w: 31.9 (16.1), *P* = .7 – EDV at 2w: 4.5 (3.9), *P* = .4	NR	– 2w: EHS, PSV, EDV, GAQ-Q1 significantly improved – 6w and 12w: SHIM, SEP-2, GAQ-Q1-2 significantly improved	– 53% in treatment group able to have an erection hard enough for penetration – 64.7% (GAQ) reported improvement in erections – 41.2% (GAQ) improved their ability to engage in sexual activity	No local or systemic complications.	2w, 6w, 12w postinjection
El-Shaer, 2021	– Median (IQR) SHIM with 100 U: 8.0 (7-9) – EHS: 1.0 (1-2) – PSV: 20 (18-32) – EDV: 6 (3-7)	– Median (IQR) SHIM with 50 U: 8.0 (8-9) – EHS: 2.0 (1-2) – PSV: 22.5 (19.5-37.5) – EDV: 6.5 (4-7.5)	– Median (IQR) SHIM with saline: 8.0 (7-9)- EHS: 1.0 (1-2) – PSV: 31.8 (20-37.5) – EDV: 6.0 (5.5-7)	NA	– Median (IQR) SHIM with 100 U at 2w: 12 (11-13), *P* < .001 – 3 m: 14 (12-16), *P* < .001 – 6 m: 14 (12-15), *P* < .001 – EHS at 2w: 3.0 (2-3), *P* < .001 – 3 m: 3.0 (3-3), *P* < .001 – 6 m: 2.0 (2-3), *P* < .001 – PSV at 2w: 33.8 (32-40), *P* = .006 – 3 m: 34 (33-40), *P* < .001 – 6 m: 37.5 (30-39), *P* < .001 – EDV at 2w: 5 (3-6.5), *P* < .001 – 3 m: 4.5 (3-5), *P* < .001 – 6 m: 3.5 (3-6), *P* < .001	– Median (IQR) SHIM with 50 U at 2w: 11 (11-12), *P* < .001 – 3 m: 13 (12-15), *P* < 0.001 – 6 m: 8 (8-10), *P* < .001 – EHS at 2w: 2.0 (2-3), *P* < .001 – 3 m: 3.0 (3-3), *P* < .001 – 6 m: 2.0 (1-2), *P* < .001 – PSV at 2w: 32 (27.5-40), *P* = .006 – 3 m: 33 (30-40), *P* < .001 – 6 m: 29 (25-38), *P* < .001 – EDV at 2w: 6 (3.5-6.5), *P* < .001 – 3 m: 5.0 (3.5-5.5), *P* < .001 – 6 m: 6.0 (4-7), *P* < .001	– Median (IQR) SHIM with saline at 2w: 8 (7-9), *P* < .001 – 3 m: 8 (5-11), *P* < .001 – 6 m: 8 (6-9), *P* < .001 – EHS at 2w: 1.0 (1-2), *P* < .001 – 3 m: 1.0 (1-2), *P* < .001 – 6 m: 1.0 (1-2), *P* < .001 – PSV at 2w: 31.5 (19-37.5), *P* = .006 – 3 m: 30 (19-37.5), *P* < .001 – 6 m: 32 (20-37.5), *P* < .001 – EDV at 2w: 7 (6-7.5), *P* < .001 – 3 m: 7 (6-7.5), *P* < .001 – 6 m: 7 (6-7.5), *P* < .001	NR	– At 2w, 3 m, 6 m SHIM, EHS, SEP-2-3, GAS-Q1-2 significantly improved (*P* < .001) in 100 U and 50 U groups vs control – Doppler findings at 2w, 3 m 6 m significantly improved (*P* < .001) in 100 U and 50 U groups vs control – At 2w and 3 m SHIM, EHS, SEP, GAS was not significantly different between 100 U and 50 U – At 6 m SHIM, SEP-2-3, GAS-Q2 was significantly higher in 100 U vs 50 U (*P* < .001)	– MCID: 4-point increase in SHIM – 40% achieved MCID	– Pain: 1 (0.8) – Priapism: 1 (0.8) – Prolonged erection during Doppler USS: 4 (3.3)	2w, 3 m, 6 m postinjection
Giuliano, 2019	– Mean (SEM) IIEF-EF for all: 13.1 (6.1) – 250 U: 12.3 (5.6)	– Mean (SEM) IIEF-EF with 500 U: 14.8 (6.6)	NA	NA	NR		NA	– Mean (SEM) increase with 250 U: 4.7 (4.5) – Mean (SEM) increase with 500 U: 5.6 (5.8)	NA	– Complete responders: IIEF-EF >26; partial responders: IIEF-EF <26 and increase in IIEF-EF vs baseline >4%-54% responders (30% complete, 24 partial)	– Penile pain: 2 (4.3)	6w postinjection

Abbreviations: BoNT, botulinum neurotoxin; ED, erectile dysfunction; EDV, end-diastolic velocity; EHS, Erection Hardness Score; FU, follow-up; GAQ, global assessment question; IIEF, International Index for Erectile Function; IQR, interquartile range; m, month; MCID, minimal clinically important difference; NA, not application; NR, not reported; PSV, peak systolic velocity; SD, standard deviation; SEM, standard error of the mean; SEP, sexual encounter profile; SHIM, Sexual Health Inventory for Men; w, week.

### Patients included

The inclusion criteria for age were *>*18 years old (yo),^13-15^^,^^18^  *>*21 yo,^16^ or 40-50 yo.^17^ All studies included patients who were unresponsive to oral PDE5i or ICI of prostaglandin-E1 (PGE1) or Trimix. All etiologies of ED were included, except for El-Shaer et al.’s study,^17^ where only vasculogenic ED was included. The median International Index for Erectile Function (IIEF-EF) score pre-BoNT-A ranged from 11.0 to 14.5 (moderate ED).

### Preparation of BoNT-A and injection technique

A penile loop, tourniquet, or rubber band was applied to the base of the penis, and 0.25-0.5 mL aboBoNT-A (250 U or 500 U), onaBoNT-A (50 U or 100 U), or incoBoNT-A (100 U)) was injected into 1-2 sites per corpus cavernosum. The penile loop or equivalent was removed after 20-30 min. The follow-up ranged from 6 weeks to 7 months.

### Effectiveness of BoNT-A

In Giuliano et al.’s 4 retrospective studies evaluating BoNT-A ICI as an add-on therapy, the first study tested 250 U or 500 U of aboBoNT-A. At 6 weeks, 54% responded, and the mean IIEF-EF increased with both doses.^18^ In their second study, both onaBoNT-A and aboBoNT-A were evaluated. The minimal clinically important difference (MCID) in the IIEF-EF score was achieved in 55.2% of patients at a median of 43.5 days, and 41% achieved MCID at a median of 5.9 months. MCID was defined as follows: mild ED, 2 points; moderate, 5 points; and severe, 7 points increase (Table 2). The type of BoNT-A did not affect the response; however, the higher 500 U aboBoNT-A dose appeared to sustain effectiveness longer compared to the 250 U dose. On multivariate analysis (MVA), only ED severity predicted response.^15^ In their third study, incoBoNT-A was evaluated, and MCID was achieved in 52% of patients at a median of 43.5 (34-71) days.^14^ In their most recent fourth study, repeated ICIs of all 3 types of BoNT-A were evaluated. The overall response rate was 77.5%, with no statistically significant difference between the 3 types of BoNT-A. The response rate increased with repeated injections: second injection, 67.5%; third injection, 87.5%, and fourth injection, 94.7%. The median duration of effectiveness was around 9 months.^13^ Only self-limiting penile pain or burn was reported as a side effect in all Giuliano et al.’s studies.

There were 2 RCTs,^16^^,^^17^ both used the Sexual Health Inventory for Men (SHIM) score, Erection Hardness Score (EHS), and Doppler USS for the assessment of pre- and postinjection of onaBoNT-A. Both RCTs demonstrated that monotherapy with onaBoNT-A improved SHIM and EHS scores as well as Doppler USS parameters compared with the control group. In El-Shaer et al.’s^17^ RCT, the MCID was achieved in 40% of patients.

### Meta-analyses

**Figure 2:**
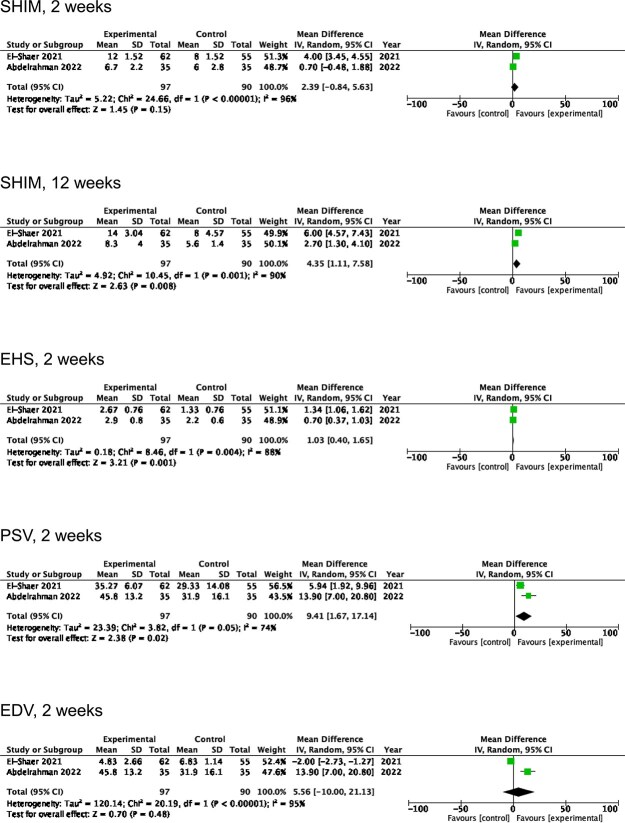
Forest plot assessing SHIM, EHS, PSV, and EDV. Abbreviations: SHIM, sexual health inventory for men; EHS, Erection Hardness Score; PSV, peak systolic velocity; EDV, end-diastolic velocity.

Only 2 studies^16^^,^^17^ included a control arm and were included in the meta-analysis (Figure 2). At 2 weeks post-ICI, there were statistically significant differences in favor of onaBoNT-A 100 U regarding EHS (mean difference, 1.03, *P* = .001) and peak systolic velocity (PSV) (mean difference, 9.41, *P* = .02). No statistical differences were observed at 2 weeks for SHIM and end-diastolic velocity (EDV). However, at 12 weeks, there was a statistically significant difference in SHIM in favor of onaBoNT-A 100 U (mean difference, 4.35, *P* = .008).

### Side effects of BoNT-A

No systemic side effects were reported. Penile pain at the injection site occurred in 0.8%-5.6% of patients and was managed conservatively. El-Shaer et al^17^ reported 1 case of priapism out of 121 patients (0.8%) at 6 months, which resolved with ephedrine ICI. The authors also reported 4 (3.3%) cases of prolonged erection during Doppler USS assessment with Trimix at 3 m, which were managed conservatively.^17^

## Discussion

In this review of 6 clinical studies, short-term results demonstrated that BoNT-A is both effective and safe in the management of ED. All 3 types of BoNT-A have been investigated as a monotherapy or as an add-on to oral PDE5i or PGE1 ICI, and all 3 toxins appeared to improve erectile function based on validated questionnaires with no significant differences in the type of BoNT-A used. However, the higher 500 U dose of aboBoNT-A seemed to have a longer lasting effect compared to the lower 250 U dose. BoNT-A appeared to be effective across all severities of ED, but a higher response rate was observed in men with mild ED. Repeated BoNT-A ICI at the patient’s request with at least a 3 month interval between injections also appeared to sustain the response. The duration of effectiveness, up to around 9 months, was similar to the duration of effect with BoNT-A intradetrusor injections for overactive bladder.^19^

Given the available data, a meta-analysis included only 2 studies, with parameters assessed in both studies being SHIM, EHS, and Doppler USS findings. Outcomes for all parameters were reported at 2 weeks post-ICI in both studies, but only SHIM was reported at 12 weeks. The results demonstrated that at 2 weeks, EHS and PSV were significantly superior in the 100 U onaBoNT-A group than in the control group. SHIM was only demonstrated to be superior in the intervention group at 12 weeks (mean difference, 4.35). Unfortunately, only 1 study reported EHS and Doppler findings at 12 weeks and 6 months; therefore, a meta-analysis of these parameters at 12 weeks was not feasible.

The 3 types of BoNT-A have the same mechanism of action, but their purification and extraction processes slightly differ. OnaBoNT-A and incoBoNT-A are similar in the doses, whereas aboBoNT-A differs, and the accepted conversion rate of ona- or incoBoNT-A to aboBoNT-A is 1:3.^20^ Physiologically, BoNT-A inhibits the release of norepinephrine from adrenergic neurons, which, in turn, may inhibit the contraction of cavernosal smooth muscle. In addition, BoNT-A inhibits the release of acetylcholine from cholinergic neurons and may block its inhibitory effect on nitric oxide synthase.^7^^,^^21^^,^^22^ With this mechanism in mind, BoNT-A should work in vasculogenic and nonvasculogenic organic causes of ED. Abdelrahman et al^16^ showed no significant differences in response rates between diabetic and nondiabetic groups. Furthermore, BoNT-A was effective in patients with spinal cord injury or who have undergone radical prostatectomy, and MVAs indicated that etiology did not predict response.^15^

Intracavernosal BoNT-A appeared to be safe, the most frequent side effect reported being penile pain associated with the injection rather than the toxin itself. Only 1 case of priapism was reported at 6 months (0.8%).

### Limitations

Data are heterogeneous with slight differences in patient characteristics, types of BoNT-A used (ona-, inco-, abo-BoNT-A), injection protocols, outcome measures (eg SHIM, IIEF-EF, EHS, and Doppler USS measures), and interval of follow-up. These variations limited the ability to perform a thorough meta-analysis. Studies without a placebo control group are not ideal for assessing effectiveness, as they may limit the validity of the findings. Future studies should standardize outcome reporting and follow-up intervals to facilitate more robust meta-analyses.

### Clinical implications

For patients who have failed pharmacotherapies or are intolerant to them and are seeking non-surgical treatment options for ED, BoNT-A may represent an evolving alternative. The ideal candidate for BoNT-A is unknown. BoNT-A has been evaluated in patients who were unresponsive to oral PDE5i or PGE1 ICI and has not been evaluated in patients as a first-line treatment. In patients who do not wish to take oral medications, BoNT-A could be tried. BoNT-A may improve the physiology of erections, but unlike treatments such as PRP, stem cells, or Li-SWT, BoNT-A does not appear to have regenerative effects. BoNT-A has been evaluated as an add-on to PDE5i and PGE1 ICI, and in the future, it would be interesting to investigate its role as a first-line treatment and in combination therapy with other regenerative treatments such as PRP or Li-SWT.

## Conclusion

Intracavernosal injection of BoNT-A has been demonstrated in a few clinical studies to be both effective and safe for short-term management of ED. However, only 2 RCTs have been conducted, featuring diverse patient groups, varying injection protocols, and restricted follow-up periods. As a result, additional RCTs are required before making definitive clinical recommendations. Nevertheless, based on current evidence, BoNT-A may become a viable non-surgical option when pharmacotherapy fails.

## Supplementary Material

Suppl_Table_1_2_Btx_ED_qfaf034
